# Older adults` sense of dignity in digitally led healthcare

**DOI:** 10.1177/09697330221095140

**Published:** 2022-06-20

**Authors:** Moonika Raja, Lisbeth Uhrenfeldt, Kathleen T Galvin, Ingjerd G Kymre

**Affiliations:** 1786 Nord University, Norway; 1947 University of Brighton, UK; 1786 Nord University, Norway

**Keywords:** eHealth, ethics, healthcare provider, older adults, Reflective Lifeworld Research

## Abstract

**Background:**

Health ministries in Europe are investing increasingly in innovative digital technologies. Older adults, who have not grown up with digital innovation, are expected to keep up with technological shifts as much as other age groups. This is ethically challenging, as it may threaten a sense of dignity and well-being in older adults.

**Research objective:**

To clarify the phenomenon of sense of dignity experienced in older adults, concerning how their expectations and needs are met within the context of digitally led healthcare in Norway.

**Research design:**

A Reflective Lifeworld Research design was chosen, and purposive, in-depth interviews were conducted.

**Participants and research context:**

The participants were 13 adults 75 years and older from Northern Norway, living at home and not receiving consistent assistance.

**Ethical considerations:**

Followed the principles of the Helsinki Declaration. This study was approved by the Social Science Data Services in Norway (project number 916119). Interviews were conducted carefully within a safe environment chosen by the participants.

**Findings:**

Older adults experience that using new digital systems in healthcare makes them become dependent with experiences of helplessness. They feel an increased sense of dependency on other people, and that recognition can assail their experience of personal dignity. Older adults not only expect digitally led healthcare to give them a feeling of safety but also experience feeling insecure concerning privacy and loss of possibilities for dialogue with healthcare providers. They are met by demands from society, which they often struggle to achieve.

**Conclusion:**

The phenomenon of sense of dignity experienced in older adults, concerning how their expectations and needs are met within digitally led healthcare, indicates a sense of feeling lost in the digital world. Further, innovative healthcare lacks focus on ethical performance. This impacts their perception of dignity, as loss of dignity is noticed especially in its rupture.

## Introduction

The European Union`s Charter of Fundamental Rights^1^ underlines the ethical basis of older adults` dignity. Health ministries in Europe are investing increasingly in innovative systems of eHealth and digitalisation.^2^ Although studies concerning digital inclusion, where healthcare is concerned, show that older citizens tend to lag behind in using and benefitting from innovative technology, older citizens in Europe are expected to embrace technological shifts just as much as other age groups.^3,4^ These papers do not refer to specific age groups; however, the generation who have not grown up with digital innovation are expected to make an effort to keep up with demanding innovations. This might challenge how healthcare providers and policy makers relate to ethics in the implementation of new technologies.

### Background

Human dignity, the fundamental human right, is inviolable and our common goal is to respect and protect it.^1^ The term dignity comes from Latin *dignitas*, appearing first in the writings of the Roman Stoics Cicero (106-43 BC) and Lucius Annaeus Seneca (4 BC-65 AD) and referring to worth and honour, values associated with being human.^5^ People refer to dignity in multiple variations and situations. In the context of this study, dignity is understood as having many resonances in human life, it is in its variations a gathering of both common value and vulnerability.^6^ Dignity is the affirmation of something valuable in oneself or another.^6^ The rights and duties which emerge from an understanding of dignity belong to each individual.^5^ Concurrently, it is an affirmation that can be ruptured, or restored in the interaction with others. It is a complex phenomenon that human beings refer to in a meaningful way.^6^ Further, removing dignity and diminishing someone`s sense of personhood is unethical.^7^ There is reference to dignity also as a core value underlying medical practice.^8^

Life expectancy in Europe has increased over the past decades and is expected to continue rising.^9^ Today, in Norway, over one in nine people are aged 70 years or more. It is assumed that roughly every 5^th^ person will be over 70 years old by 2060.^10^ Observers argue that ageing in Europe has implications for many sectors of society, including healthcare, providing a stimulus for developing innovative sustainable goods and services adapted to the needs of older citizens.^9,11^

Nordic countries are internationally recognised as leading the way in the innovative practice of digitally transforming older adults` healthcare to meet the challenges that all European countries are facing.^12^ However, there is still a need to be vigilant about the ethical aspects of and the impact on the sense of human dignity inherent in this digital transformation.

The history of Norwegian eHealth goes back to 1997 when the government published the eHealth strategy ‘More health for each bIT–information technology for a better health service’,^13^ and has been developing since. The digitalisation strategy in Norway, between 2017 and 2022, aims for all citizens to have safe and easy access to healthcare services.^12^ A digitalised patient journal should be available in the Norwegian national eHealth portal. Including all the information about one`s health, access to booking doctor`s appointments, easy access to E-consultations, E-prescriptions and information about available healthcare services.^14^ These are elements which, hereinafter in the text, fall under the term digitally led healthcare.

Ethical principles that may affect human rights are important in healthcare.^15^ Beauchamp and Childress^16^ underline four of them: autonomy, beneficence, justice and nonmaleficence. In addition, avoiding harm, honesty, loyalty, privacy and utility are overall ethical principles in healthcare.^17,18^ A recent integrative review study about ethical issues in eHealth use, found that important ethical aspects are privacy, justice, trust, beneficence and nonmaleficence.^19^ Older citizens in Europe have faced ethical issues such as trust, privacy and beneficence when using innovative technology. Older adults from Austria, France and Hungary were confronted with an ethical issue, in being concerned that the homecare robot`s camera might not respect their privacy.^20^ Older people in Sweden faced the ethical issue of trusting the digital pen eHealth device because the users were too weak to use the tool by themselves, or it was not working.^21^ It was found among older adults and their carers in Sweden using digital technology within homecare that the system was not comparable with benefits of nursing home.^22^ Likewise Zsiga et al.^20^ conclude, as might be anticipated, that homecare robots cannot replace people, which is an important human factors consideration with inherent ethical aspects.

Studies from Norway covering digital technology and including older people, focus on special technological solutions or cover only specific groups of people, such as individuals with a physical disability or cognitive impairment.^23–25^ In Southwestern Norway, it was found that among adults 70 years and over, with a physical disability, self-efficacy significantly reduced their ability to use smart house technology and that earlier experience with devices had an effect on technology perception.^23^ Older people in Norway consider technology as more useful for other older adults than for themselves, or as useful in the future.^24^ Research conducted in Oslo about welfare technology, that included some older persons, suggested that systems that worked in the first time, may not work later since their health is in constant change and the function of technology is changing over time as well.^26^ The studies cited from Norway about technology and eHealth systems, including older adults, do not address dignity or other ethical issues such as privacy, justice or trust.^23–26^

Skär and Söderberg^27^ underline the importance of preserving patients` dignity and autonomy when using eHealth systems in healthcare. Preserving human dignity in the face of demanding digital innovations is a challenge and may impact older persons` individual experiences. In order to utilise future technology and digitally led healthcare adapted to the needs of older adults in a dignified way, one must first determine how existing solutions impact their sense of dignity. This study is part of a wider research^28^ addressing barriers to, and facilitators of, societal digital demands in older adults.

### Aim

This study clarifies the phenomenon of sense of dignity experienced in older adults, concerning how their expectations and needs are met within the context of digitally led healthcare in Norway.

### Method

#### Theoretical framework

Since in this study dignity is understood as having many resonances in human life, it is important to illuminate contextual variations, which a phenomenological inductive perspective opens up for. The research design is framed by a phenomenological lifeworld perspective and Reflective Lifeworld Research (RLR), as described by Dahlberg et al.^29^ The RLR has epistemological and ontological roots in the phenomenology of German and French philosophers.^29^ Lifeworld is seen as the *horizon* of one`s experiences, from which it is not possible to withdraw.^30^ One is ‘being-in-the-world’ (*in-der-Welt-sein*), not merely in it spatially, but *belongs there,* and has access to the lifeworld through one`s body.^31,32^ The aim of RLR is to describe the studied phenomenon as it is experienced by the participants. The phenomenological perspective helps to understand dignity from the value of relations, and as such it is suitable for this study.

#### Recruitment

A purposive sample of 13 older adults, 75 years or older, was recruited for individual interviews. The participants were Norwegian citizens living at home and not receiving consistent assistance. The flexible pension age in Norway is between 62 and 67 years, so 75 years marks about 10 years from their transition into retirement. This age-group has spent recent years away from the workplace digital transition and may not have experienced digitalisation in the way younger adults have.^33^

Participants were recruited from a local voluntary association established in their home city in Norway in 1996. It is based on the University of the Third Age, an organisation founded in France in the 1970s.^34^ The first author first wrote to and then met with its committee. A gatekeeper from the organisation, who was not part of the research team, shared information to individuals who met the inclusion criteria.^35^ To avoid any coercion, no contact was made with potential participants until they had been fully informed about the project.

#### Data collection

Data were gathered through individual in-depth purposive interviews in autumn 2020. It was during the second wave of Coronavirus SARS-CoV-2, and national infection control measures were in place. As little social contact as possible was recommended.^36^ The interviews were held according to local infection control regulations. The participants were asked to describe their everyday experiences concerning technology, including digitally led healthcare in Norway. Contextual variations in their daily life were important as these variations of meaning in their experiences are helpful in seeing the phenomenon`s structure.^29^

The interviews were carried out by the first author. They were conducted to explore: current experience of using digital technology, expectations and needs about digitally led healthcare in Norway. The interviews started with an opening question: ‘*When I say the word “technology” what comes to mind for you?’* The participants were asked if they had ever used assistive devices and digital solutions offered by the healthcare system in Norway. In accordance with Dahlberg et al.,^29^ probing questions were asked to obtain details, to gather descriptions of examples and to clarify unclear statements. Information about participants` age, gender, educational level and the experience from usage of digital tools was gathered.

#### Data analysis

The RLR analysis^29^ was undertaken to clarify the essential characteristics of the phenomenon as faithfully as possible. RLR has lifeworld as a starting point in understanding meaningful experience. The meaning of an expression relates to the person and the context in which it is being expressed.^37^ The essences are their phenomena and the phenomena are their essences, everything is experienced as something, and the essence cannot be separated from the phenomenon that it is the essence of. With this approach, the researchers in this study tried to uncover the phenomenon while staying open to participant`s expressed meaning of their lifeworld.^29^

Following Dahlberg et al.,^29^ the entire collection of interview texts was initially read to get a sense of a whole, and the parts were always seen in the terms of the whole. The descriptions were divided into units of meaning and meanings that seemed to belong to each other were temporarily put together in clusters by first and last author. These clusters were analysed in looking for intentionality and organised, then related to each other while looking for essential meanings that described the phenomenon. The constituents were formed inductively within a reflective understanding of intentionality in the participants’ descriptions. The research process led to a new written understanding of the phenomenon`s essential meaning described in terms of essence. Essence is uncovered by identification of its constituents, the meanings that constitute the actual essence.

In this study, the interviews were analysed with an open attitude from the researcher in terms of *bridling*. *Bridling* characterises a shift from a natural attitude into a phenomenological attitude of openness.^29^ *Bridling* involves being present and questioning one`s own understanding rather than taking it for granted. Gradually evolving one`s understanding, neither randomly nor too quickly.^38^ The authors had their own preunderstandings overlaying their experience and different from those of the respondents, overlaying their lifeworlds. In this study, these preunderstandings were set aside through a kind of bridling, to allow the essential meaning of the phenomenon to stand out, without limiting the openness of the research.

#### Ethical considerations

This study was firstly approved (project number 916119) by Social Science Data Services in Norway (NSD). The study was, secondly, scrutinised by INNOVATEDIGNITY`s Ethical Scrutiny and Advisory Board to assure its ethical quality and ability to contribute to the European Union`s Horizon 2020 open access initiatives.^35^ The data material was treated confidentially and anonymised according to the guidelines of the Norwegian National Committee for Research Ethics in the Social Sciences and the Humanities^39^ and General Data Protection Regulation.^40^ The research team did not contact potential participants until they had received written and oral information about anonymity and the possibility of withdrawing at any time. Ethical principles were considered since particular situations or characteristics of a person could place them as vulnerable and at risk of harm.^41^ Safe environment was chosen by the participants, some had also a family member present during the interview. Information about purpose of the study, recording and data treatment was repeated immediately before interview and all participants gave written, informed consent before participation. The interviews were conducted carefully, following risk assessment, the interviewer paid close attention to the participant`s reactions during the interview to avoid distress and allowed extra time.

### Results

This section shares the participants` (see Table 1) experiences about dignity in digitally led healthcare in Norway.

**Table 1.** Information about the participants.

No	Gender	Age	Marital status and residence	Experience with eHealth
1	Female	75	Lives alone in an apartment	Many E-consultations with healthcare providers, some use of E-prescriptions, little use of eHealth portal
2	Female	75	Lives with spouse in a private house	Regular use of E-prescriptions, little use of eHealth portal
3	Female	77	Lives with spouse in a private house	Regular use of eHealth portal and E-prescriptions, some digital bookings of doctor`s appointments
4	*Male*	79	Lives with spouse in a private house	Little use of eHealth portal
5	Female	79	Lives with spouse in a private house	Some digital bookings of doctor`s appointments
6	Female	79	Lives alone in an apartment	Very little use of eHealth portal
7	Female	80	Lives with spouse in a private house	Some E-consultations with healthcare providers
8	*Male*	81	Lives with spouse in a private house	Some use of E-prescriptions, digital bookings of doctor`s appointments (with help)
9	Female	81	Lives with spouse in a private house	Reads sometimes notices from healthcare providers in eHealth portal
10	Female	82	Lives alone in an apartment	Some digital bookings of doctor`s appointments, some use of eHealth portal and E-prescriptions
11	*Male*	82	Lives with spouse in a private house	Have tried to use eHealth portal (did not succeed)
12	*Male*	82	Lives with spouse in a private house	Regular use of E-prescriptions
13	Female	89	Lives alone in a private house	Have tried to use eHealth portal (did not succeed)

#### The essential structure of meaning

Older adults experience that using new digital systems in healthcare leads to them becoming dependent and gives rise to a sense of helplessness. They feel an increased sense of dependency on other people and that, in turn, assails their experience of personal dignity. Older people expect that digitally led healthcare will give a sense of safety, but it also produces a feeling of insecurity concerning privacy and loss of the relational aspect of having a productive dialogue with healthcare providers and being treated as individuals. Participants are subject to demands from society in terms of being expected to know how to use systems, and are thrown into situations where they have to acquire new skills promptly, which they often struggle to achieve.

Below, the constituents of the essential structure of the phenomenon are described (Table 2).

**Table 2.** Constituents and the essential structure of meaning.

Constituents	The essential structure of meaning
Becoming dependent of help	Older adults experience that using new digital systems in healthcare leads to them becoming dependent and gives rise to a sense of helplessness. They feel an increased sense of dependency on other people and that, in turn, assails their experience of personal dignity. Older people expect that digitally led healthcare will give a sense of safety, but it also produces a feeling of insecurity concerning privacy and loss of the relational aspect of having a productive dialogue with healthcare providers and being treated as individuals. Participants are subject to demands from society in terms of being expected to know how to use systems, and are thrown into situations where they have to acquire new skills promptly, which they often struggle to achieve
Importance of human contact and being treated as individuals	
Prerequisites to develop new skills while being thrown into unfamiliar digital world	
Insecurity concerning privacy in the digital world	
Paradoxically technology can give a feeling of safety	

#### Becoming dependent on help

Older people describe becoming dependent on help due to experiencing obstacles when using digital technology. Loss of independence in healthcare context, that is continuously more digitised, can make them vulnerable and affect their experience of dignity. The participants describe experiencing obstacles, such as lack of basic knowledge and instructions, rapid changes in the program, technology not working and missing access to the systems. Having difficulties with booking doctor`s appointments, checking test results or using videoconferencing with healthcare providers. *‘And what it is for me and my generation. It is that we have mastered our things. We have been able to do what we should be able to. And suddenly we are so illiterate about it’.* Another participant shared experience of using eHealth system: *‘Knowledge has come so fast. That we, who were older, we did not know about it until it was actually expected that we should be able to use all those systems’.* The participants have experienced that they are rendered passive and need help when expected to use new digital systems. They describe a sudden change from independence to becoming dependent on other people in the digital world impacting their feelings negatively. *‘I do not want to ask for help. That does not feel good’.*

#### Importance of human contact and being treated as individuals

The importance of human contact and as such being treated as individuals, affects the experience of dignity. The need to hear human voice was expressed like this: *'I do not send an email to my doctor. I take my phone and I call. And I can hear a human being in the other end, and I ask for an appointment. And he asks what is the reason for coming. I feel like I need that’.* It is important to *hear* the other person`s voice and feel someone`s presence.

There are concerns about reduced human contact in healthcare systems and fear of what lies ahead*. ‘It is completely different to sit and talk to a doctor than to just get it with a robot voice in a way. So I think some of this is scary going forward’.* Another participant said: *‘ I am very skeptical of that replacement. How far can you get with replacing human contact with digital solutions? I do not find peace about it. What will there be offered by the health system in a few years?’* They expect that one does not make it so complicated that the human contact, and thereby being treated as individuals, disappears in the future.

#### Prerequisites to develop new skills while being thrown into unfamiliar digital world

The participants express that they are not immersed naturally but thrown into the digital world in a disruptive way and that affects their sense of dignity. They describe that the technological language and different icons in use in digital solutions are strange and alien to them. *‘Very little language for us, which is simple enough to understand. But yes. It is not easy. They speak their language but it is not language that those who are not computer educated would understand’.* A word that has one meaning in everyday life can have a totally different meaning in technological terms, and understanding all those does not come naturally.

They describe that using digital technology is a demanding and overly rushed process. There are expectations towards older adults to have skills that they struggle to achieve and that affects their experience of dignity. Persons need to have time and take it at an appropriate tempo and have the possibility to try system out. People are different and need individual approaches. It is also important to write down the process step by step or get a manual and have follow-up guidance after a while. Experiences from using eHealth systems were: *‘It is not like we learn it right away but someone comes back and asks how is it going and if you understand it. Then there must be tight follow-up’.* Another participant said: *‘You have to do it yourself and have to say what you do and then you have to take notes because you forget too quickly. It takes time. So I would not forget it when they are gone. So it is very important that you also get a manual about it’.* Struggling constantly with an unfamiliar process can be humiliating and diminish agency and therefor impact the sense of dignity.

#### Insecurity concerning privacy in the digital world

The participants express that they lack information about eHealth systems, how these systems are used in healthcare and who has access to their personal data. Different concerns about lack of privacy affect their sense of dignity. On the one hand, that programmers’ take over and user loses control. *‘To think, where is the one who sits somewhere and controls it. Who overrides it and makes it more difficult?’* On the other hand, the participants express having little information about what kind of data will be in the system about their health and medical history. Unanswered questions about ethical issues concerning privacy were raised by the participants: Who, exactly has access to the information and when? What can their medical data be used for? Is it somehow possible to use the information against them? They do expect privacy about their health issues. *‘I wish, it was still possible to have something that is private. That not everything is posted online and that there is still private room between a doctor and a patient’.* They feel that their privacy is exposed if it is included in data, but at the same time, technology can also give a feeling of safety. This range includes varying degrees of emphasis from the extreme to everyday securities and insecurities.

#### Paradoxically technology can give a feeling of safety

Having the possibility of contacting the health services if something happens and being followed up makes persons feel safe. That can help to enhance the feeling of being worthy and thereby affect sense of dignity. Most of the participants had experienced that some of their close ones or themselves had a feeling of safety when using a safety alarm. Safety alarms were offered by the health system in cooperation with the local health service centre. A participant said: *‘Once I came downstairs and I fell on that chair. And I thought that now I have to use an alarm. For now I do not know how to stand… and a lady came and helped me’.* If the person knows how to use eHealth systems or a digital aid, it is seen to be useful and helpful. For example, a safety alarm was considered easy to use and as giving a sense of safety.

### Discussion

The phenomenon of sense of dignity experienced in older adults, in how their expectations and needs are met within digitally led healthcare, indicates a sense of being bewildered in the digital world. To preserve human dignity in demanding digital innovations is challenging and includes facing ethical issues such as dependence, privacy, humility, vulnerability, need for human contact and being treated as individuals. The scope and importance of eHealth have increased in recent years and will continue to be an essential part of healthcare delivery. The essential key finding is that older people have a sense of becoming dependent in terms of situational helplessness when using new digital healthcare systems. The need to ask for help and a sense of failure may make older adults feel more vulnerable and even humiliated. This unfamiliar state may impact their sense of personal dignity, as dignity`s path of loss is through vulnerability.^7^ Preserving human dignity is therefore ethically important to older citizens, and reference is often made to dignity as a core value underlying medical practice.^8^

Older people are often met with demands from society in terms of being able to acquire new skills promptly. The ever-evolving nature of technology means that one needs ever increasing levels of digital literacy, to be able to trust the new systems and maintain a sense of inclusion. They are a heterogenous group with regard to their digital technology use because their motivation, past employment and existing knowledge vary.^42^ Those who participated in this research, do often struggle to achieve the levels of digital literacy needed. They must make an effort to learn new strategies or must depend on others. That might categorise them as somewhat vulnerable, potentially humiliated through impeded agency and thereby diminish in their sense of dignity. These potential consequences accord with studies of older adults needing educational support to be included in the digital society.^43,44^ Similarly, a study about older adults` attitudes to eHealth in primary healthcare found that there is a need for a strong focus on information and support for older citizens concerning digital demands and eHealth.^45^

Older people express differences between quick and general help, and assistance that really helps to understand eHealth and improve digital skills. Achieving a new skill may increase the benefits of the new system and may impact the perception of dignity in a positive way. Younger adults are found to perform significantly better and learn more quickly than older adults.^46^ Older adults underlined the importance of having the possibility of trying out the devices more than once by themselves, having enough time to contemplate and most importantly, having a manual to use when needed. In terms of learning, there are age-related differences and similarities in cerebellar and cortical brain function.^46^ In addition to age-related differences to learning, the participants shared experiences about manuals that they did not understand because of unfamiliar icons and difficult technological language. It is important not only to offer help but make sure that assistance is suitable in order to improve older adults` digital skills. That can impact their independence and experience of dignity in a positive way.

The feeling of insecurity concerns the lost possibility for direct dialogue with healthcare providers. Likewise the contrast with the traditional healthcare delivery shows a lack of in-person face-to-face contact that digital healthcare seems to miss. Use of eHealth has the potential to misrepresent or represent incompletely the human aspect of medical communication.^47^ The feeling of insecurity of being treated as individuals may impact older adults` perception of dignity. On the one hand, concerns arise about healthcare providers having the information they need to make well-grounded clinical decisions when they obtain information through technologies. The ethics of digitally led healthcare should not focus on the patient as a collection of images or a data set but as a whole person.^47^ Nurses using eHealth solutions are challenged in certain situations to not take the focus in healthcare away from the patient, they should ensure that care remains person-centred.^48^ On the other hand, the participants expressed a need for in-person conversations, which underlines how they value the relational aspect and human contact to be treated as individuals. A previous study from Norway about use of welfare technology also claims that reducing social contact as a result of technology is something that worries healthcare providers.^26^ In-person contact is important and may not be the same as when replaced by contact through technology. This may impact older adults` perception of dignity, as the understanding of dignity belongs to each individual.^6^ It is underlined that nurses should ensure that technological devices should not replace human relationships.^48^

In this study the experiences of older people revealed a feeling of insecurity concerning privacy when using digital health systems. According to Kuziemski et al.^49^ privacy issues are a major concern in eHealth systems, but the risk and extent of privacy issues differ according to the pattern of eHealth being used. It is important to be aware of these issues and have guidance on how to manage them. Some older citizens in Europe have also experienced concerns about eHealth systems, especially cameras, respecting their privacy, but conversely, others were much less worried about their privacy than researchers had assumed.^50^ Older adults need more information about the systems, about who has access to their data and about how to use the systems safely. This can help to reduce hesitancy concerning privacy when using digital health systems and thereby help to improve persons’ sense of dignity.

An expectation that digitally led healthcare will give a sense of safety in terms of human support when needed is strong. Safety includes achieving physical, social and psychological security, rights to be protected and fulfilled under international human rights.^1^ That kind of security can improve older adults’ well-being. Safety and dignity are interconnected in caring for older people.^51^ Suitable help to use new technology and support through eHealth can provide safety and thereby improve older persons perception of personal control and security have impacts on a sense of dignity. It is important that older people are consulted in the extent of digitally led healthcare. Nurses should ensure that the use of technology is compatible with the safety, dignity and rights of users.^48^ In this way future technical developments can also contribute to an emphasis on preserving agency and has relevance of dignity.

#### Strengths and limitations

Strength of this study was that the interviews uncovered rich and various data and gave new knowledge about the phenomenon. Also that the request for participation was not-binding, and participants were given the opportunity to avoid complicated travelling, which did not exclude anyone for this reason. The number of participants is considered sufficient and both genders were represented. Interviews were back translated to the original language and checked for accuracy. Credibility was addressed by two authors analysing the results.

It is a limitation that participants in this study were able to leave their homes and attend meetings at the local older adults` centre. Further research is needed into older citizens staying mostly at home and possibly experiencing functional limitations and other constraints due to their situation.

### Conclusion

The phenomenon of sense of dignity experienced in older adults, concerning how their expectations and needs are met within digitally led healthcare, indicates a sense of feeling lost in the digital world and this might be ethically challenging. It impacts their perception of dignified ageing. Loss of dignity by being thrown into a situation in this way is unethical and especially noticed in its rupture. Rupture because of being put into an unfamiliar world, need for human contact and insecurity concerning privacy in the digital world. The results of this research show that if older adults are better informed about technologies and provided with the necessary digital skills, they see digitalisation more as an opportunity. There is a spectrum of variations of emphasis, on the one hand unfamiliar technology makes you insecure and increases a sense of vulnerability and lacks focus on ethical considerations when innovating healthcare digitally. On the other hand, digitalisation gives a sense of safety and opens up new opportunities. The phenomenological perspective helps us to understand dignity from the value of relations and how it can be ruptured in terms of being bewildered in the digital world. This tension illuminates ethical challenges that healthcare providers and policymakers should acknowledge for future innovations.
